# *LGR5*, *HES1* and *ATOH1* in Young Rectal Cancer Patients in Egyptian

**DOI:** 10.31557/APJCP.2021.22.9.2819

**Published:** 2021-09

**Authors:** Heba Morsy, Ahmed Gaballah, Mohamed Samir, Vandrome Nakundi, Mohamed Shamseya, Hanan Mahrous, Abeer Ghazal, Mervat Hashish, Waleed Arafat

**Affiliations:** 1 *Department of Human Genetics, Medical Research Institute, Alexandria, Egypt. *; 2 *Department of Microbiology, Medical Research Institute, Alexandria, Egypt. *; 3 *Department of Clinical and Experimental Surgery, Medical Research Institute, Alexandria, Egypt. *; 4 *Department of Clinical Oncology and Nuclear Medicine, Faculty of Medicine, Alexandria University, Egypt. *; 5 *Department of Clinical and Experimental Internal Medicine, Medical Research Institute, Alexandria, Egypt. *; 6 *Comprehensive Cancer Center of Alexandria (CCC Alex), Alexandria, Egypt. *

**Keywords:** Rectal cancer, Egypt, Young adults, Cancer stem cells, Gene expression, LGR5, HES1, ATOH1

## Abstract

**Objective:**

The study aimed to delineate the gene expression profile of *LGR5*, *HES1* and *ATOH1* in young Egyptian rectal cancer (RC) patients and investigate the correlation between expression profiles and clinical outcome.

**Methods:**

The study was conducted on 30 young Egyptian RC patients. Expression study of *LGR5*, *HES1* and *ATOH1* were performed by quantitative PCR (QPCR) based on comparative Cq method after normalization to adjacent non tumor tissues and ACTB as a reference gene. Patients were followed up for assessment of response to neoadjuvant chemoradiotherapy (CRT) based on revised RECIST1.1.

**Result:**

The study detected overexpression of *LGR5* and *HES1* and down-regulation of *ATOH1* in human RC tissues compared to non- tumor tissues. High expression of *LGR5* was correlated with more depth of tumor invasion, lymph node (LN) metastasis, advanced cTNM stage and mesorectal fascia (MRF) involvement. More prominently, high *LGR5* expression level was associated with poor response to CRT. *LGR5* was suggested as unfavorable prognostic biomarker for RC. Conversely, *HES1 *and *ATOH1* expression did not show significant association with most of the studied clinical criteria nor response to CRT. Still, *HES1* and *ATOH1* were significantly and inversely associated with presence of mucinous component.

**Conclusion:**

High *LGR5* expression is indicative of poor prognosis among young Egyptian RC patients and is proposed as a predictive marker of resistance to neoadjuvant CRT. However, *HES1* and *ATOH1* expressions were not prognostic nor predictive of response to CRT. Overall, *LGR5*, *HES1* and *ATOH1* gene expression patterns among young onset RC patients, are in line with patterns encountered in older age groups.

## Introduction

In Egypt, Colorectal cancer (CRC) is the sixth most common cause of cancer-related deaths, with a relative frequency of 4.2 % (Ibrahim et al.,2014). The percentage of young-onset CRC cases in Egyptians is strikingly high with more than one third of cases occurring under 40 years (Soliman et al., 1997; Soliman et al., 1999). Different molecular pathology and unique pathogenesis features of CRC in Egyptian compared to Western patients, was reported (Soliman et al., 2001). Since, more than half of the Egyptian population is younger than 50 years, a mounting public health problem of CRC among young adults in Egypt should be highly considered (Silla et al., 2014).

Despite extensive CRC-focused research over the past 20 years, the clinical outcome of CRC is still very poor (Siegel et al., 2015). A growing number of research proposed cancer stem cells (CSCs), as the main element contributing to tumor progression, relapse, metastasis and therapeutic resistance (Visvader et al., 2008). A thorough understanding of the specific biomarkers and the signaling pathways governing the functions of CSCs is crucial for identification of valuable prognostic markers and therapeutic targets for CRC (Chen et al., 2013).

**Figure 1:**
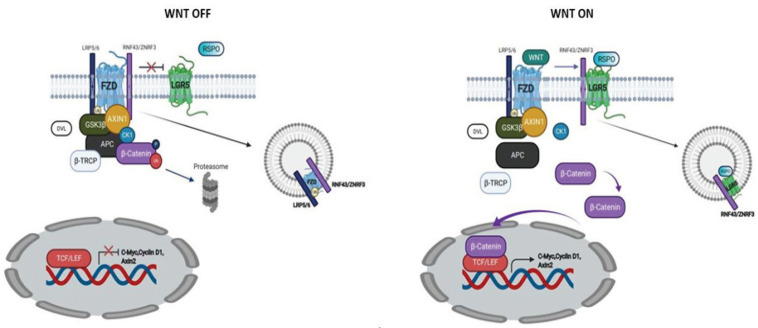
WNT OFF, Without LGR5/RSPO Complex, Two Transmembrane E3 Ligases (RNF43 and ZNRF3) Remove the Wnt Receptors from the Cell Membrane, Internalize and Degrade them. WNT ON, The binding of RESPO to LGR5 neutralize the transmembrane ligases, they cannot remove the Wnt receptors from the cell surface. FZD and LRP5/6 binds Wnt ligands leading to stabilized β-catenin

*LGR5* (Figure 1), also known as GPR49, was proposed as the most selective and promising marker of CSCs in CRC (Wu et al., 2012; Barker et al., 2014). Some studies, used the Immunohistochemistry technology to detect its expression, have shown the correlation between the *LGR5* upregulation and the histopathological characteristics of CRC. Furthermore, the same results have been addressed by meta-analysis studies (Chen et al., 2014; Han et al., 2015; Jiang et al., 2016). However, these studies are limited by the fact that the presence of antibodies targeting the *LGR5* remains in doubt (Barker et al., 2014; Kemper et al., 2012; Baker et al.,2015).

The studies, detected the *LGR5* using in situ hybridization in CRC, have found different results; same of them have shown correlation between *LGR5* upregulation and poor prognosis (Baker et al.,2015; Uchida et al., 2010) while others did not find any association (Ziskin et al., 2013; Jang et al., 2018). Thus, further research, using alternative method, is required as most previous studies have been carried out in elderly patients. 

Tumors consist of distinct CSC populations with different biomarkers that the association with the histopathological features of CRC has still needed to be assessed (Nimmakayala et al., 2019). The upregulation of CD133 has been associated with metastasis and distant recurrence of CRC after chemoradiotherapy (Yasuda et al., 2009; Horst et al., 2009; Saigusa et al., 2010). The CD24 has shown controversial result, its cytoplasmic expression demonstrated correlation with invasion and differentiation but not association with reduced patient overall survival (Choi et al., 2009; Ahmed et al., 2009).

**Figure 2:**
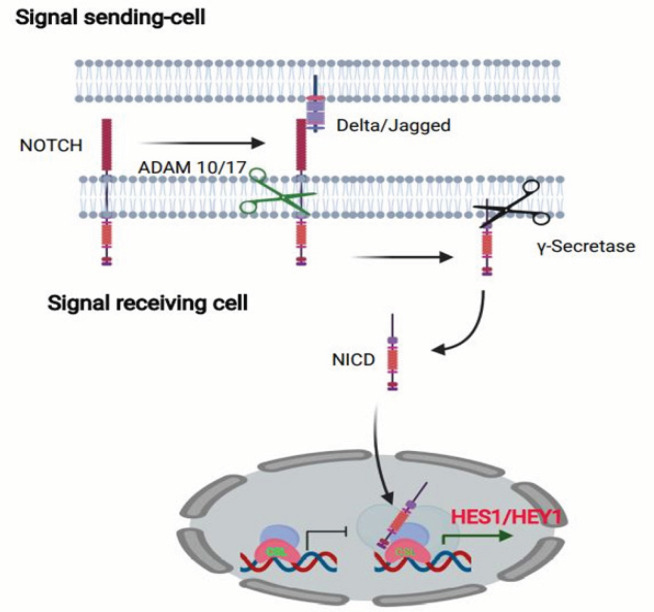
Notch Signaling and the Maintenance of CSC. Two Nearby cells, one signal sending and other signal receiving cell interact. The binding of Delta/Jagged to the Notch leads to S2 cleavage by ADAM10 or 17, which is followed by S3 cleavage by γ-secretase. The S3 cleavage gives rise to an intracellular Notch fragment (NICD) that migrates into the nucleus where it binds to a complex of protein, leading to the de-repression of transcription of hair/enhancer of split (Hes) and Hey

Notch signaling (Figure 2) is implicated in maintenance of CSCs and its aberrant activation plays a key role in carcinogenesis and progression of human malignancy. Several studies have revealed how Notch signaling pathway might regulate *HES1* and *ATOH1* expressions in normal and cancerous tissues; moreover it worth mentioning that same studies have shown that the inhibition of Notch signaling reduces the *LGR5* intestinal stem cell population (Srinivasan et al., 2016). 

The CSCs hypothesis raises questions regarding current diagnostic and therapeutic modalities (Vaiopoulos et al., 2012). In spite of remarkable and non-stop advances in studying CSCs in CRC, further molecular characterization is crucial especially in Egypt, where molecular aspects of CSCs have not been fully investigated among Egyptian patients.

The aim of the current study was to delineate the gene expression profile of three CSCs related genes, *LGR5*, *HES1* and *ATOH1*, among young Egyptian rectal cancer patients, and investigate possible association between these genes’ expression and clinical outcome, including response to neoadjuvant chemoradiotherapy.

## Materials and Methods

*Study Participants*

The study was conducted on 30 young Egyptian RC patients (less than 40 years old) with histopathologically confirmed adenocarcinoma. Patients were recruited from Gastroenterology and Endoscopy Unit at Alexandria Main University Hospital and Medical Research Institute within the period from August 2013 to February 2015. The study was conducted in accordance with the guidelines of the Declaration of Helsinki and all participants signed an informed consent before participation in the study.

*Methods*

All patients enrolled in the study were thoroughly examined. Paired tumor and non-tumor adjacent mucosal tissues were obtained from patients by routine biopsy techniques during colonoscopy followed by histopathological examination. C.T abdomen & pelvis, pelvic MRI were requested for adequate clinical staging of RC according to TNM staging, 7^th^ edition. Patients received neoadjuvant CRT comprising 3D conformational radiotherapy using CT planning to deliver a dose of 45-50.4 Gray with conventional fractionation every day except Thursday and Friday for 5-7 weeks, concurrent with daily Capecitabine (825g/m^2^ Bid from D1- D5/ week). 

Evaluation of the change in tumor burden is considered a valuable feature of the clinical assessment of cancer therapeutics. As a consequence, the revised RECIST 1.1 guidelines were adopted in the current study as a primary response endpoint to neoadjuvant CRT (Eisenhauer et al., 2009). Patients were followed up and reassessed for response to neoadjuvant therapy after 6 weeks, then categorized according to RECIST guidelines 1.1 into 4 groups; complete response (CR), partial response (PR), stable disease (SD) and progressive disease (PD). 

*Gene expression study by qPCR*

Paired tumor and non-tumor adjacent tissue samples were immediately fixed in RNA later stabilization reagent. Total RNA isolation from all tissue samples was carried out using RNeasy Protect Mini Kit (Qiagen, Germany) according to the manufacturer’s instructions. The concentration of RNA was determined by measuring the absorbance at 260 nm (A_260_) by Nanodrop (Thermo Scientific, USA). Reverse transcription of RNA to cDNA was done using TaqMan Reverse Transcription Reagents (Invitrogen, USA) according to manufacturer`s protocol. PCR products were amplified from cDNA samples using the SYBR Green Universal Master Mix (Life technologies, USA), in addition to specific primers for target genes *LGR5*, *HES1*, *ATOH1* according to previously published sequences (Kobayashi et al., 2012; Chen et al., 2010; Kong et al., 2012). QPCR reactions were done in triplicate for all samples and performed in Step one real time PCR (Applied Biosystems). Relative expression of target genes was calculated using the comparative Cq method (2^–ΔΔCq^) by normalization to ACTB as a reference gene and adjacent non- tumor tissue as calibrators (Livak et al., 2001).

*Statistical Analysis*

Statistical analysis was done using IBM SPSS statistics program version 21 and Medcalc program. Mann-Whitney test (MW) was used to detect significant difference in the median quantitative variables between two groups of patients. Kruskal Wallis test (KW) was done to compare the median quantitative variables between the several categories of depth of tumor invasion. For significant results, pair wise comparison was done using adjusted p value by Bonferroni correction. 

Receiver operating characteristic (ROC) curve analysis was done to detect the diagnostic accuracy of different indices for response to treatment. Area under the curve (AUC), sensitivity, specificity, positive predictive value and negative predictive value were used to evaluate each index (Leeflang et al., 2013). All statistical tests were done at 0.05 significance level.

## Results

The present study was carried out on 30 young Egyptian patients with RC adenocarcinoma who were eligible for neoadjuvant therapy. Demographic and clinical criteria of the studied patients in addition to rectal tumor characteristics, including response to neoadjuvant CRT, are summarized in Table 1.

**Figure 3:**
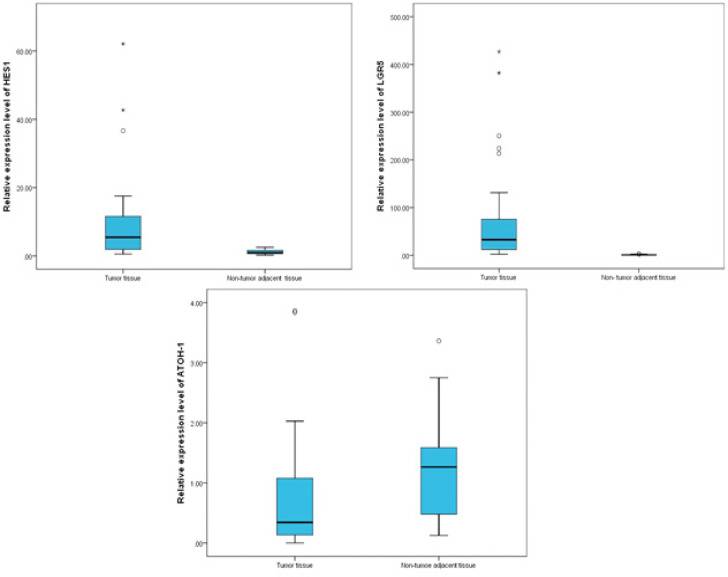
Box Plot Showing Expression of LGR5, HES1& ATOH1 in Rectal Tumor and Non-tumor Adjacent tissues. The upper and lower borders of the box represent 25^th^ and 75^th^ percentile, the horizontal line inside the box represents the median (50^th^). Circles represent the outliers. Data were analyzed using Mann-Whitney test

Relative quantitative expression of *LGR5*, *HES1* and *ATOH1* in rectal tumor tissues compared to paired non-tumor adjacent tissues revealed significant overexpression of *LGR5* and *HES1* (p <0.001). Conversely, *ATOH1* was significantly down regulated in tumor tissues relative to non- tumor adjacent tissues (p= 0.05) (Figure 3).

**Figure 4:**
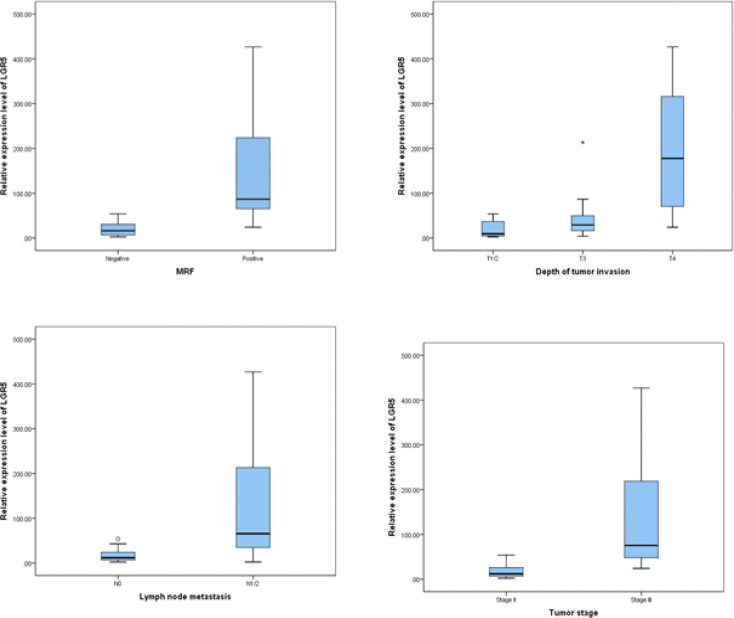
Correlation between LGR5 Expression Levels and MRF Involvement, Depth of Tumor Invasion, LN Metastasis and Clinical Tumor Staging

The median *LGR5* fold change was significantly correlated to MRF involvement among the studied rectal tumors (p <0.001) (Figure 4). A substantially higher levels of *LGR5* expression were noted in tumors with involved MRF compared to those with free MRF. Likewise, statistically significant difference was detected in median *LGR5* expression level along different staging parameters. Particularly, *LGR5* expression levels significantly correlated with depth of tumor invasion (p=0.002). Post hoc paired comparisons revealed that median expression levels of *LGR5* in cT4 was significantly higher than in cT1/2 (adjusted p =0.002) and cT3 (adjusted p= 0.031). However, *LGR5* expression levels didn`t show significant difference between cT1/2 and cT3 (adjusted p= 0.520). Furthermore, Expression of *LGR5* was significantly higher in patients with regional LN metastasis compared to patients without LN metastasis (p <0.001). Consequently, stage III rectal tumors showed highly significant overexpression of *LGR5* compared to stage II. (p <0.001). On the contrary, no significant correlations were observed between relative *LGR5* expression and patients demographic characteristics (age and sex), tumor location nor pathological features (Table 2, Figure 3).

*HES1* expression levels in distal rectal tumors ranged from 0.59 to 42.67 with a median of 8.28, while in proximal rectal tumors it ranged from 0.6 to 62.05 with a median of 3.39. Hence, tumors in Lower 2/3 of rectum had significantly higher *HES1* expression levels than those in Upper 1/3 (p=0.019). Statistical analysis revealed no significant correlation between *HES1* relative expression and pathological grade of differentiation (p = 0.402). Still, a statistically significant association was identified between *HES1* relative expression levels and presence of mucinous component in the studied rectal adenocarcinomas (p= 0.022). The median *HES1* fold change in rectal tumors with mucinous components was significantly lower than non- mucinous tumors. On the other hand, no significant difference was observed in *HES1* expression levels as regards MRF involvement (p= 0.28) and different staging parameters, including cT (p= 0.749), cN (p= 0.103) and cTNM staging (p=0.233). Additionally, median *HES1* fold change didn`t show statistically significant difference with RC patients` demographic characteristics (Age and sex) (Table 2).

*ATOH1* median fold change was significantly lower among RC patients under 30 years old compared to older patients aged 30-40 years (p=0.037). Though, no significant difference in expression levels of *ATOH1* was noted as regards sex of the patients (p= 0.51) nor the location of the rectal tumors (p= 0.95). Furthermore, rectal tumors with different pathological grades of differentiation did not show statistically significant difference in *ATOH1* expression levels (p=0.95). Conversely, presence of mucinous component in rectal adenocarcinomas was a highly significant factor correlating with *ATOH1* fold change, where median *ATOH1* expression level was significantly higher in rectal adenocarcinomas with mucinous components compared to non-mucinous tumors (p <0.001). Finally, no statistically significant difference was observed between *ATOH1* fold change and MRF involvement (p =0.103), depth of tumor invasion (p =0.158), spread to regional LN (p =0.536) nor clinical staging (p=0.217) of rectal tumors (Table 2).

Correlation analysis revealed highly significant association between *LGR5* expression levels and response to CRT among the studied RC patients (p < 0.001). *LGR5* was substantially over expressed among non- responders (SD & PD) to CRT as it ranged from 24.04- 426.87 with a median of 70.58. On the contrary, responders (CR &PR) to CRT showed much lower levels of *LGR5* expression, ranging from 2.36 to 53.73 with a median of 11.58. Nevertheless, response to neoadjuvant CRT was not associated with *HES1* nor *ATOH1* relative expression levels (p=0.498, p=0.142, respectively) (Table 3, Figure 3).

**Figure 5:**
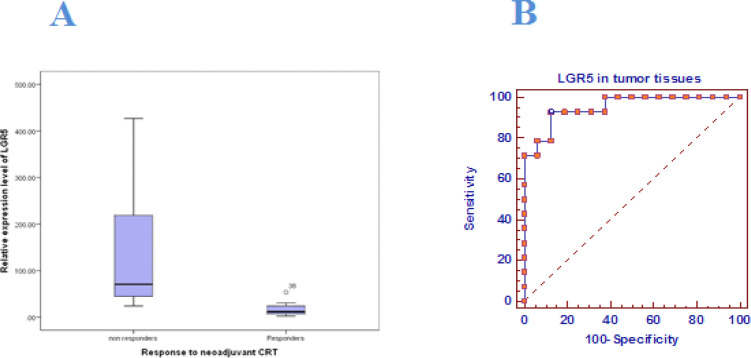
Response to Neoadjuvant CRT Based on RECIST 1.1 Guidelines in Relation to LGR5 Relative Expression among the studied RC patients. A, Box plot showing LGR5 over expression among non- responders to CRT compared to responders. B, ROC curve analysis indicating that LGR5 expression is an excellent predictive factor for response to neoadjuvant CRT

Moreover, ROC curve analysis for expression of *LGR5* and response to neoadjuvant CRT showed that *LGR5* expression can significantly discriminate between responders and non- responders by 95 % accuracy (AUC=0.95, p <0.001). This indicated that the *LGR5* expression level is an excellent predictive factor for response among RC patients with high sensitivity (92.86 %) and specificity (87.5 %). A cut-off value of 30.65 best distinguished between responder and non- responder RC patients (Figure 5).

**Table 1:** Demographic and Clinical Characteristics of RC Patients

Characteristics	No. (%)
Sex	
Male	17 (56.7)
Female	13 (43.3)
Age	
20- 30 years	11 (36.7)
30-40 years	19 (63.3)
Clinical presentation	
Bleeding per rectum	21 (70)
Pain and tenesmus	13 (43.3)
Constipation	12 (40)
Mucous in stool	4 (13.3)
Rectal tumor site	
Upper 1/3	12(40)
Lower 2/3	18 (60)
Tumor grade	
Low grade	6 (20)
High grade	24 (80)
Adenocarcinoma with mucinous component	
Present	7 (23.3)
Absent	23 (76.6)
MRF involvement	
Positive	13 (43.3)
Negative	17 (56.7)
Clinical staging	
cT	
cT1/2	8 (26.7)
cT3	14 (46.6)
cT4	8 (26.7)
cN	
cN0	13 (43.3)
cN1	11 (36.7)
cN2	6 (20)
cM	
cM0	30 (100)
cTNM	
stage II	15 (50)
stage III	15 (50)
Response to neoadjuvant CRT based on RECIST 1.1 guidelines	
CR	8 (26.7)
PR	6 (20)
SD	6 (20)
PD	10 (33.3)

MRF, Mesorectal fascia; CR, complete response; PR, Partial response; SD, Stable disease; PD, Progressive disease

**Table 2:** Correlation between LGR5, HES1& ATOH1 Genes Fold Change and Clinicopathological Criteria of the Studied RC Patients

Clinicopathological criteria							
		LGR5 fold change	Statist. sig.	HES1 fold change	Statist. sig.	ATOH1 fold change	Statist. sig.
		Median (min-max)		Median (min-max)		Median (min-max)	
Age	<30 y	53.73 (2.36- 382.06)	^MW^p= 0.471	4.45 (0.59-42.67)	^MW^p= 0.35	0.17 (0.002 -1.68)	^MW^p= 0.037*
	30- 40 y	27.81(4.11- 426.87)		5.59 (0.6-62.05)		0.74 (0.02- 3.86)	
Sex	Male	30.65 (2.36- 382.06)	^MW^p= 0.536	5.59 (0.59-62.05)	^MW^p= 1.00	0.21 (0.00- 3.86)	^MW^p= 0.51
	Female	34.7188 (11.22- 426.87)		5.41 (1.44- 36.64)		0.38 (0.10- 2.03)	
Tumor location	U 1/3	36.84 (2.41- 426.87)	^MW^p= 0.723	3.39 (0.60- 62.05)	^MW^p= 0.019*	0.29 (0.002- 3.86)	^MW^p= 0.95
	L 2/3	32.68 (2.36- 250.32)		8.28 (0.59- 42.67)		0.45 (0.02-2.03)	
Pathological grade	Low	20.93 (4.11- 75.46)	^MW^p = 0.251	6.89 (3.26- 62.05)	^MW^p = 0.402	0.17 (0.03- 0.85)	^MW^p= 0.95
	High	40.59 (2.36-426.87)		5.22 (0.59- 42.67)		0.45 (0.002-3.86)	
Mucinous component	Present	65.24 (6.86- 250.32)	^MW^p= 0.288	1.44 (0.59- 13.5)	^MW^p= 0.022*	1.68 (1.10- 3.86)	^MW^p <0.001*
	Absent	30.65 (2.36- 426.87)		5.6 (0.89- 62.05)		0.21 (0.002- 1.08)	
MRF	MRF-	16.42 (2.36- 53.73)	^MW^p <0.001*	5.6 (0.89- 62.05)	^MW^p= 0.28	0.21 (0.02-1.16)	^MW^p= 0.103
	MRF+	86.68 (24.04- 426.87)		5.05 (0.59- 36.64)		0.85 (0.002- 3.86)	
cT	cT1/2	9.39 (2.36- 53.73) ^a**^	^KW^p= 0.002*	7.25 (0.89- 42.67)	^KW^p= 0.749	0.18 (0.02- 1.16)	^KW^p= 0.158
	cT3	29.23 (4.11- 213.43) ^a**^		4.96 (0.59- 62.05)		0.41 (0.002-1.68)	
	cT4	177.71 (24.04- 426.87)^b**^		6.62 (0.6- 36.64)		0.88 (0.09-3.86)	
cN	cN0	11.94 (2.41- 53.73)	^MW^p <0.001*	8.37 (1.55- 62.05)	^MW^p= 0.103	0.52 (0.02- 1.16)	^MW^p= 0.536
	cN1/2	65.69 (2.36- 426.87)		5.05 (0.59- 36.64)		0.31 (0.002- 3.86)	
cTNM	Stage II	11.94 (2.36- 53.73)	^MW^p <0.001*	6.75 (0.89- 62.05)	^MW^p= 0.233	0.21(0.02- 1.16)	^MW^p= 0.217
	Stage III	75.46 (24.04- 426.87)		5.05 (0.59- 36.64)		0.38 (0.002- 3.86)	

MWp, p value for Mann Whitney test; KWp, p value for Kruskal Wallis test; *, Statistically significant at p ≤ 0.05 ; **, Medians with differing superscripts within columns are significantly different at the adjusted p ≤ 0.05 based on post hoc paired comparison.

**Table 3:** Relative Gene Expression in RC Patients in Relation to Response to Neoadjuvant CRT

Relative gene expression	Responders	Non-responders	Statist. Sig.
LGR5	11.58 (2.36- 53.73)	70.58 (24.04- 426.87)	p < 0.001*
Median (Min-Max)			
HES1	5.28 (0.59- 36.64)	5.50 (0.89- 62.05)	p = 0.498
Median (Min-Max)			
ATOH1	0.45 (0.002- 3.86)	0.19 (0.02-1.16)	p = 0.142
Median (Min-Max)			

p, p value for Mann Whitney test. *, Statistically significant at p ≤ 0.05.

## Discussion

The current work studied gene expression of 3 CSCs related genes, namely *LGR5*, *HES1*, and *ATOH1* in rectal adenocarcinomas among young Egyptian patients. 

*LGR5* median expression level in rectal tumor tissues was significantly higher than paired normal tissues (Table 1). Increased protein and mRNA expression of *LGR5*, using qPCR or Immunohistochemistry (IHC), have been previously reported in CRC tissues compared to normal mucosa. (Uchida et al., 2010; He et al., 2014; Kleist et al., 2011; Hsu et al., 2013) Overexpression of *LGR5* in CRC maybe attributed to enrichment of ‘stem-like’ cancer cells, upregulation of the Wnt signaling pathway and/or Wnt -dependent maintenance of stemness (Uchida et al., 2010, Walker et al., 2011). It was suggested that transformed *LGR5*-positive stem cells are the origin of cancer in the intestine (Hsu et al., 2013; Yui et al.; 2012).Therefore, *LGR5* was proposed as an ideal marker for CSCs in CRC (Kemper et al., 2012; Hirsch et al., 2014).

On the other hand, few studies put forward the opposite points, where increased invasion and tumorigenicity, besides enhanced Wnt signaling were observed upon knockdown of *LGR5* in a xenograft model (Walker et al., 2011). This remark is in agreement with the other data suggesting *LGR5* as a negative regulator of canonical Wnt pathways (Garcia et al., 2009; Wu et al., 2014). Such discrepancy suggests that *LGR5* and its ligands R-spondin might have opposing roles in different contexts which remain to be elucidated. Another possible explanation is that these studies observed a negative feedback loop where *LGR5* expression keeps in check over-activation of canonical Wnt signaling (Walker et al., 2011). Moreover, the discrepancies between different studies, could be attributed also to the fact that *LGR5* expression can be, alternatively or additionally, stimulated by other mechanisms than Wnt signaling (Sancho et al., 2009; Tanese et al., 2008). Another hypothesis of relation between *LGR5* expression and Wnt pathway was proposed by two reports, which supported a bi-modal regulation of *LGR5* expression by Wnt signaling: induction of *LGR5* at medium levels of Wnt activation but loss of expression with higher levels of Wnt activation (Sun et al., 2009; Lewis et al., 2010). 

The present study next investigated *HES1* gene expression as a marker for Notch signaling. Results displayed that *HES1* mRNA levels were significantly elevated in rectal tumor relative to normal tissues (Table 1). Comparable results of *HES1* mRNA overexpression were observed by several studies (Jin et al., 2012; Yuan et al., 2015; Candy et al., 2013; Meng et al., 2007). In primary CRC, Notch signaling was shown to be strongly activated, and has an important role in cancer initiation and progression (Qiao et al., 2009).

The current work studied, as well, the expression of ATOH (Kong et al., 2001). *ATOH1* mRNA expression was significantly downregulated in rectal tumor tissues compared to normal tissues, with very low levels of *ATOH1* expression noted among most of malignant tissues (Table 1). A growing number of studies further confirmed our results and supported a tumor suppressive function of *ATOH1*( Leow et al., 2005; Park et al., 2006; Bossuyt et al., 2009). It was suggested that tumor suppressive function of *ATOH1* is mediated by its effects on CSCs (Kazanjian et al., 2010). In CRC, several mechanisms work to skew the normal Notch-*ATOH1* balance to favor Notch activation, including silencing of *ATOH1* by genetic and epigenetic mechanisms (Bossuyt et al., 2009). 

In the current study, correlation analysis demonstrated that *LGR5* expression in human RC clinical specimens was significantly correlated with depth of tumor invasion, LN metastasis, cTNM stage and MRF involvement, but it was not correlated with gender, age, tumor site, nor pathological features (Table 2). Our results suggest that high expression levels of *LGR5* are correlated with more malignant tumors. This is in agreement with a meta-analysis by Jiang et al., 2015 who reported that *LGR5* overexpression is significantly associated with deep invasion, LN metastasis, distant metastasis and AJCC stage, however, it was not correlated with tumor grade (Jiang et al., 2016). Their results proposed *LGR5* expression as a prognostic factor for CRC patients. Correspondingly, Han et al.,2015 meta-analysis, showed almost equivalent results (Han et al., 2015). Nevertheless, Yang et al., 2016 meta-analysis, detected correlation between *LGR5* expression and TNM stage only (Yang et al., 2016). Studying the association of *LGR5* with clinical outcome in RC after preoperative CRT, Saigusa et al.,2012, reported also that elevated *LGR5* expression was significantly correlated with poor recurrence-free and Overall Survival (Saigusa et al., 2012). Other groups had concluded that *LGR5* expression was involved in the CRC progression, as well (Uchida et al., 2010; Hsu et al., 2013, Ong et al., 2016). 

The most well established correlations with *LGR5* expression encountered in the aforementioned studies (Uchida et al., 2010; Han et al., 2015; Wu et al., 2016) are in accordance with our results. Overall, these findings indicate that *LGR5* gene expression association with clinical findings among young onset RC patients, are in line with gene expression patterns encountered in previous studies conducted in older age groups.

On the other hand, Ziskin et al., (2013) showed that *LGR5* expression, was not associated with increased tumor aggressiveness and was not a prognostic factor for CRC. Surprisingly, it was also reported that methylation of CSCs associated Wnt target genes predicts poor prognosis in CRC patients, hence high *LGR5* expression is associated with a favorable prognosis (De Sousa et al., 2011). 

According to our results, MRF involvement was positively correlated with *LGR5* expression level. (Table 2). Involvement of or close proximity to the MRF preoperatively increases the risk of compromised Circumferential Resection Margin (CRM) after surgery, which was repeatedly defined as an independent predictor of a poor outcome.(Quirke et al., 2009, Park et al., 2014). Hence, such correlation with high *LGR5* expression is not unexpected and in line with the main assumption of unfavorable impact of high *LGR5* expression. 

On the other hand, no correlations were found with gender and pathological features according to the current work. Previous study suggested that the role of *LGR5* might be different between male and female patients in tumorigenesis (Hsu et al., 1998). However, previous meta-analysis, Han et al., (2015) showed that *LGR5* was not obviously correlated with the gender of patients. Regarding relation to pathological criteria, previous results are inconsistent (Wu et al., 2012; Jiang et al., 2016; Jiang et al., 2015; Yang et al., 2016).

 Correlations between *LGR5* expression in primary tumors from CRC patients and different clinicopathological features are inconsistent. Many factors may have influenced such discrepancy in results. For example, additional subgroup analysis in previous meta-analysis, (Chen et al., 2014) revealed that many factors influenced the significance of the correlation between *LGR5* expression and prognosis in CRC patients. Hence, the discrepancy in the correlation results could be attributed to relatively small number of patients in some of the studies including the present study, and the dissimilarity of patient populations in different studies. It was observed that high *LGR5* expression was significantly associated with poor outcome in the studies conducted on Asian patients, but not in non- Asians (Chen et al., 2014; Jiang et al., 2015).Therefore, further investigations were recommended to verify whether the prognostic value of *LGR5* in CRC is associated with the variation of the study population. This observation further elucidates the importance of our research on Egyptian patients. The diversity of techniques used to detect expression of *LGR5* maybe another factor contributing to heterogeneity of results in different studies. Most of the studies primarily depend on IHC, which is highly altered by methodological factors. Another potential mechanism to the opposing results is variants of the *LGR5* gene (Kleist et al., 2012; Szkandera et al., 2015). 

Aiming to explore the clinical significance of Notch signaling in RC, the possible relation between *HES1* and *ATOH1* expression levels and various clinicopathological characteristics of rectal tumors were studied to evaluate their prognostic potential. *HES1* and *ATOH1* expression levels did not show significant association with most of the clinicopathological criteria of the studied RC patients. Yet, both *HES1* and *ATOH1* expression levels were significantly correlated with presence of mucinous component in rectal adenocarcinomas (Table 2).

Many studies have recently focused on the role of Notch signaling pathway in influencing the differentiation decision of cells in the GIT. A previous study agreed with our results, where overexpression of Notch transcription factors, including *HES1* was not associated with tumor differentiation (Candy et al., 2013). Nevertheless, other studies indicated that the expression levels of *HES1* were associated with the pathological tumor type and degree of differentiation (Jin et al., 2012; Gao et al., 2014; Ahadi et al., 2016). As regards to *ATOH1*, a study on mesenchymal GIT tumor was concordant with our results (Huang et al., 2014). 

Clinical study of *HES1* overexpression in CRC have shown that the *HES1* expression was not associated with survival (Reedijk et al., 2008). In accordance with our results, activation of Notch signaling was noted in human CRC, but the level of signaling is not prognostic. These findings were further confirmed later (Candy et al., 2013). Nevertheless, other studies showed contradictory results, where high *HES1* expression was associated with better or poor prognosis (Yuan et al., 2015; Ahadi et al., 2016). Although *HES1* is considered the most well characterized downstream target of Notch pathway, *HES1* can also be elevated via other signals (Ingram et al., 2008; Stockhausen et al., 2005). This may partly explain the inconsistent results on the prognostic significance of *HES1* in previous studies. In addition, the diverse heterogeneity of tumor samples included in different studies may further add to discrepancy in results. Therefore, the association and prognostic value of *HES1* in CRC remains to be investigated in future studies.

Regarding the association of clinical findings with *ATOH1* expression, the present work didn`t find significant correlation between *ATOH1* mRNA levels and various tumor criteria (Table 2). A number of studies have suggested that loss of *ATOH1* strongly enhances the formation and progression of different types of tumors and was identified as independent predictive factors for poor outcome (Han et al., 2015; Huang et al., 2014). In CRC, because the expressed *ATOH1* protein is degraded, its function has not been elucidated in details since most of studies relies on IHC analysis. 

Mucinous cancers (MC) are distinct classes of CRC, with unique genetic and epigenetic alterations (Song et al., 2005; Bosman et al., 2010). MC may exhibit more aggressive behavior and a worse prognosis than their non-mucinous counterparts. (Yamamoto et al., 1993). In the present study, *HES1* and *ATOH1* were inversely expressed in rectal adenocarcinomas with mucinous component (Table 2). Previous studies have demonstrated that cell fate in human gut epithelium seems to be directly controlled by two main Notch target genes: *HES1* and *ATOH1* (Fre et al., 2005). Hence, the pattern of clinical expression noted in our results is consistent with molecular understanding of Notch signaling. Results of animal studies revealed that intestines of Math1 (mouse homolog of human *ATOH1*) null mice fail to develop secretory cell lineages (Yang et al., 2001). The role of *ATOH1* in mucinous cancer is far beyond just induction of differentiation. Microarray analysis showed attainment of more malignant potential by *ATOH1* protein stabilization, suggesting the mechanism by which MC are more malignant than non-mucinous adenocarcinoma (Kano et al., 2013).

In the present study, gene expression data showed that patients with lower levels of *LGR5* expression had significantly better response than patients with higher levels of *LGR5* expression (Table 3). An evidence on the predictive power of *LGR5* expression was demonstrated by previous research showing better response to 5-FU based treatment among patients with low *LGR5* level compared to those with high level. This finding agrees with our study, although in different patient cohort, since our patients received Capecitabine (5FU based treatment) (Hsu et al., 2013). Furthermore, it was reported that RC specimens from patients with poor pathological response had significantly higher *LGR5* expression levels than those exhibiting a positive response after CRT. Thus, it was suggested that *LGR5* expression may be implicated in resistance to CRT in RC (Saigusa et al., 2012).

On the contrary, Planutis et al 2015, (Planutis et al., 2015) in their work hypothesized that *LGR5*-expressing cells would be more chemotherapy sensitive, as *LGR5* is usually a marker of dividing cells. They concluded that CRC cells that express *LGR5* are more sensitive to the chemotherapeutic compounds Irinotecan and Oxaliplatin, but not to 5-FU. They proposed that *LGR5* makes cells vulnerable to chemotherapy by increasing their propensity to divide through activation of the Wnt/β-catenin pathway. Since genetic polymorphism in *LGR5* gene was suggested as predictive biomarker for response to FU in CRC patients, variants of *LGR5* gene is one factor which might explain this discrepancy between different studies (Szkandera et al., 2015).

The molecular mechanisms underlying *LGR5*-associated chemoresistance were investigated by Liu et al 2013 (Hsu et al., 2013). They illustrated that elevated *LGR5* caused resistance to 5-FU and Oxaliplatin, which was associated with high ABCB1 expression, an efflux pump for chemotherapeutic drugs. The limited success of chemotherapy may be also due to the failure of current therapies to effectively kill CSCs. Remarkably, it was reported that after anticancer agent treatment, some fast-proliferating *LGR5*-positive CSCs converted to slow proliferating *LGR5*-negative cells and entered quiescence to escape chemotherapy-mediated killing. After the drug removal, these *LGR5*-negative cells reverted to the *LGR5*-positive state to reconstitute the entire tumor, suggesting a pool of CSCs with the ability to interconvert between two distinct states (Kobayashi et al., 2012). The authors proposed that the ability of CSCs to switch between these two states might explain how some rare CSCs survive during drug therapy (Kobayashi et al., 2012).

Altogether, these notions support our observation that high *LGR5* expression level was associated with resistance to neoadjuvant CRT in young RC patients, and that early onset RC did not show difference in the role of *LGR5* as a predictive marker of chemoresistance. Therefore, *LGR5* is an attractive target for combating chemoresistance in RC.

Given its role in cellular proliferation and CRC tumorigenesis and progression, the present study investigated whether tumor expression of Notch target genes, *HES1* and *ATOH1*, may be used as predictors of response to CRT in RC patients. However, no statistically significant correlation was detected between *HES1* nor *ATOH1* and response to CRT. (Table 3). In CRC, few studies have addressed the role of HES1 in response to chemotherapy. It was shown that 5-FU, Oxaliplatin and Irinotecan-induced chemoresistance in CRC cells was promoted by Notch transcription factors, such as *HES1*, and abrogated using Notch inhibitory therapy using Gamma -Secretase Inhibitors (GSI) (Meng et al., 2007). A clinical study, reported that the combination of HEY1, *HES1* and SOX9 protein overexpression were predictive of poorer response to chemotherapy in CRC patients (Candy et al., 2013). As the included cohort of patients in our study didn`t show different responses to CRT in relation to different *HES1* expression levels, this may be due to lack of enough samples or the variation in age and site of tumors in our patients. Hence, additional studies seem to be needed especially comparing young and old RC patients in regard to the correlation between *HES1* expression and chemoresistance.

The role of *ATOH1* in chemoresistance in CRC patients was the point of investigation in a few recent studies. It was revealed that cells transducing *ATOH1* showed chemoresistance against Oxaliplatin. Interestingly, it was reported that Oxaliplatin stabilized *ATOH1* protein, resulting in the suppression of the apoptosis signaling. Induction of CSCs and mucinous phenotypes, by *ATOH1* protein stabilization, leading to chemoresistance, was described, as well. Another interesting result was that the *ATOH1* protein extended the G0/G1 phase of the cell cycle and led to avoidance of G2 phase entry, which most of alkylating agents target (Kano et al., 2013). These results are in concordant with findings observed in a later study (Fukushima et al., 2015). Collectively, these findings illustrated the acquisition of chemoresistance by MC that express the *ATOH1* protein. However, almost all these studies were conducted on MC. Unfortunately, our study didn`t follow this model as our cohort included both mucinous and non-mucinous rectal adenocarcinomas, with only few tumors showing mucinous components. Hence this might have hindered attainment of significant results on *ATOH1*expression relation to response to CRT. 

In conclusion, the current study suggested that *LGR5* may not only serve as a novel prognostic indicator in young RC patients, but may also be an excellent predictor for response to CRT. Additionally, the molecular characterization of rectal adenocarcinomas with mucinous components revealed high *ATOH1* expression levels, adding supplementary evidence to its critical effect on cellular differentiation. However, *HES1* and *ATOH1 *expressions were not prognostic nor predictive of outcome or response to CRT. Overall, our findings indicate that *LGR5*, *HES1* and *ATOH1* gene expression patterns among young onset RC patients, are in line with patterns encountered in older age groups. It is noteworthy that the current study is, to the best of our knowledge, the first Egyptian study to address the molecular features of young RC patients by studying CSCs related genes. 

However, data in this study should be interpreted with some caution, because the baseline characteristics of patients might have affected the conclusions of each of the studied genes, including the sample size, follow-up period and clinical stage, among other aspects. Hence, a larger study population, inclusion of both young and old Egyptian RC patients, with a long-term follow-up is needed to validate these results. Besides, putting in consideration the known predictive and prognostic factors, such as MMR deficiency, is highly suggested. One more limitation may be due to the functional redundancy of many signaling molecules and the strong interaction between signaling pathways which have diverse effects on downstream gene expression. 

Finally, the CSCs hypothesis may herald a paradigm shift in oncologic diagnosis and treatment. We are only beginning to understand the multifaceted roles of CSCs in RC. Further studies will reveal more detailed mechanisms by which CSCs contribute to intestinal tumor progression, hence better characterize RC molecular prognostic markers. Research directed towards identification and treatment of CSCs, may eventually lead to better screening, early detection, treatment, and prognosis of RC patients.

## Author Contribution Statement

1. Waleed Arafat- conceived, designed the experiments and acquired the funding. 2. Heba Morsy, Ahmed Gaballah, Mohamed Samir- did the data collection. 3. Heba Morsy, Mohamed Shamseya, Hanan Mahrous, Abeer Ghazal, Mervat Hashish. -contributed to specific areas of the methods, data analysis, statistics, and quality control. 4. Waleed Arafat, Heba Morsy- analyzed the data 5. Vandrome Nakundi, Heba Morsy: wrote the first draft of the manuscript and conceived the diagrams. All authors read and approved the final manuscript.
